# Tobacco Use Patterns and Associated Factors Among Youth in Kampala’s Informal Settlements: A Cross-Sectional Study in Bwaise

**DOI:** 10.21203/rs.3.rs-6682635/v1

**Published:** 2025-05-20

**Authors:** Nakitende Joyce, Anthony Kirabira, Adelaine Aryaija-Karemani, Nazarius Mbona Tumwesigye

**Affiliations:** 1. Department of Epidemiology and Biostatistics, School of Public Health, Makerere University; 2. Medical Research Council/ Uganda Virus Research Institute and London School of Hygiene and Tropical Medicine Uganda Research Unit.; 3. Department of Health Policy and Planning, School of Public Health, Makerere University

**Keywords:** Tobacco use patterns, youth, informal settlements, daily tobacco smoking, smokeless tobacco use, *kibanga*

## Abstract

Tobacco use among the youth in Uganda remains higher than general national estimates. To tackle this challenge, the Centre for Tobacco Control in Africa and the World Health Organization assert a need for current data and tobacco use monitoring with emphasis on most at-risk populations. In Uganda, routine monitoring is done through quinquennials; Uganda Demographic Health Survey, World Health Organisation stepwise surveys, Global Adult Tobacco Survey and Global Youth Tobacco Survey. Although recent assessments reported tobacco use among the general population, current data is scarce among the youth in informal settlements. We sought to assess the patterns of tobacco use and associated factors among the youth in informal settlements, with focus on daily tobacco smoking and smokeless tobacco use.

**Methods:**

We used a cross-sectional study design. Secondary data which was collected between March 2021 and April 2021 among 422 youth aged 18–30 in Bwaise was used. We used STATA version 17.0 to analyse the data, and Modified Poisson regression with robust standard errors to assess for associations.

**Results:**

The daily smokers were 160/422 (37.9%) while the smokeless tobacco users were 69/422 (16.4%). ‘*Kibanga*’ was the most smoked product (145/160) daily. Being male (adj.PR=1.68 [95%CI=1.27–2.23]), aged 21–30 years (adj.PR=1.72 [95% CI=1.28–2.33]), below secondary education (adj.PR=0.69[95%CI=0.56–0.87]), from central Uganda (adj.PR=0.64[95%CI=0.46–0.89]), not knowing that smoking causes serious illness (adj.PR=1.5[95%CI=1.11–2.03]), heart attacks (adj.PR=1.49[95%CI=1.11–4.83]) and lung cancer (adj.PR=1.71[95%CI=1.25–2.35]) were significantly associated with daily tobacco smoking. Age 21–30 years (adj.PR=1.75 [95%CI=1.04–2.96]), not believing that smokeless tobacco causes heart attacks and serious illness (adj.PR=3.02 [95%CI=1.87–4.87]) were associated with smokeless tobacco use.

**Conclusion:**

Daily tobacco smoking and smokeless tobacco use prevalence were both higher than the national estimates. Future policy interventions among among youth in informal settlements should target males, aged 21–30 years, of education level below secondary, originating from central Uganda, as well as address knowledge gaps on the dangers of tobacco use

## Background

The World Health Organization (WHO) reports that tobacco use has increased by two-fold in the last four decades, among the youth and the poor, Sub-Saharan Africa (SSA) inclusive (1, 2). This is attributed to its budding population, augmented consumer buying power, and penetration of markets by the tobacco industry (1). Most current smokers started in adolescence and continued with the habit to adulthood (3).

In Uganda, tobacco use among the youth exceeds estimates from the general population (4, 5), but data among youth in informal settlements is scarce. To tackle this challenge, the Center for Tobacco Control in Africa (CTCA) asserts a need for local evidence including: current epidemiology of tobacco use among most at-risk populations, risk factors for tobacco use, current patterns and trends of tobacco use, exposure to old and new products, who is most at risk and the drivers to use of these products (6). By 2016, 1 in 10 persons used any tobacco products daily in Uganda (7, 8). Unfortunately, the risk of developing non-communicable diseases (NCDs) increases with frequency and amount of tobacco use, putting daily users at a high risk (9, 10).

In a survey among the youth in Bwaise, by the Community Anti-Drug Coalitions of America (CADCA) international program; 47.5% of the youth said they would try using cigarettes or other tobacco products if given an opportunity, implying that tobacco use is a problem in Bwaise, but the current patterns and associated factors are not documented.

Tobacco use in Uganda is monitored through the quinquennial Uganda Demographic Health Survey (UDHS), the Uganda Household Survey (UHHS), the Global Adult Tobacco survey (GATS), the Global Youth tobacco survey (GYTS) and the NCD stepwise risk factor surveys; which are however general and lack data specific to the youth in informal settlements.

Most studies which specifically assessed tobacco use in informal settlements were outside Uganda (11–14), others assessed substance use (15–17), while others were conducted among high school students, and are outdated (18, 19). Moreover, new tobacco products have emerged such as smokeless tobacco (SLT) whose prevalence data are inconsistent (20, 21). In this study, we aimed to assess the current patterns of tobacco use and associated factors among the youth in informal settlements in Kampala, using Bwaise as a case study, with focus on daily tobacco smoking and smokeless tobacco use.

## Methods

### Study design and population

This was a cross-sectional study, secondary data from a previous study was used. Youth aged 18–30 years who were residents of Bwaise informal settlement in Kampala, were included. According to the constitution of the Republic of Uganda, a youth is any person between the ages of 18 to 30 years (22)

### Sample size and precision

There were 422 observations in the database, the precision of the study was estimated based on the 422 observations using the Kish Leslie formula for proportions (23), $n=\frac{z^{2}pq}{e^{2}}$ Where n =422, Z=1.96, P=50%, q =1-p, e was the desired level of precision. Substituting in the formula, $422=\frac{1.96^{2}X0.5(1-0.5)}{e^{2}}$, e =0.05.

### Description of the primary study

The primary study was cross-sectional and utilized mixed methods, it was conducted within Bwaise, the largest informal settlement in Kampala, the capital city of Uganda. It is divided into 3 parishes; one (Parish 111) was selected randomly. It had six zones and each was represented in the study sample. The first household was selected randomly. The rest were sampled systematically at an interval of 16. The data was collected between March 2021 and April 2021 using a semi-structured questionnaire adapted from the GATS and the WHO stepwise approach, both are standardized instruments for global use.

### Description of the data set

There were 422 participant observations and 66 variables from the primary study database. The Independent Variables were the socio-demographics (gender, age, number of school years, religion, marital status, origin, education level, main work status, number of people older than 18 years in the household, monthly income) and Knowledge. The Dependent Variables were daily tobacco smoking and smokeless tobacco use

### Data analysis

We imported primary data from Epi data entry version 3.0 into STATA version17. Inconsistencies, impossible differences, duplications and missingness were checked, variables with missingness of more than 50% were excluded.

At univariate level, we described the socio-demographics, the patterns of tobacco use and knowledge-as one composite based on the GATS instrument (24, 25). At bivariate level, cross tabulations were done. Variables whose p-values were less than 0.2 or plausible were included in the multivariate models. Daily smokers were compared to a combined group of occasional smokers and non-current smokers. Similar categorizations were done in other studies (26–29)

At multivariate level, we used Variance Inflation Factor (VIF) to rule out multi-collinearity. We used modified Poisson regression with robust standard errors because the probability of both outcomes was above 10% and they were binary, (Logistics regression inflates the odds ratios if the outcome is above 10%). We fitted separate models for each outcome. Variables whose p-value was less than 0.05 were considered significant. We used Akaike Information Criteria (AIC) and Bayesian Information Criteria (BIC) to select the best model against other competing models. The model with the lowest AIC and BIC for each outcome was selected

### Ethical considerations

Approval to conduct this study was sought and obtained from the Makerere University School of Public Health Research Ethics Committee (MaKSPH-REC), protocol number 141. Secondary data was used, the need for informed consent was waived. The data was anonymized to maintain confidentiality.

## Results

### Characteristics of the study participants

There were 422 participants in the study, 248 (58.8%) were male. The mean age was 23 (±3.64) years while the majority (70.8%) were aged 21–30 years. The average school years was 9.6 (±3.27) while each household had on average 3 (±2) people older than 18 years. Most participants (68%) had secondary or university-level education and 82.7% were employed

### Tobacco use patterns among the youth in Bwaise informal settlement, Kampala, Uganda.

The mean age at the start of tobacco use was 17 years (±3.91). Current smokers were 222/422 (52.6%). Almost three-quarters, 160/222 (72.1%) of the current smokers do it daily, this accounts for 37.9% (160/422) of the entire sample. Less than one-quarter (16.4%, 69/422) of the sample used smokeless tobacco.

### Types of tobacco products used by the youth in Bwaise informal settlement.

Almost three-quarters (71.3%) smoked ‘*kibanga*’ (a local term used to mean a mixture of tobacco and marijuana) daily followed by manufactured cigarettes (45.6%). Kuber (43.5%) was the most used smokeless tobacco product, followed by mijaj (26.1%). Most participants used more than one tobacco product, therefore the (n) don’t add up to (n=222) and (n=69).

### Knowledge of the effects of tobacco use on health among the youth in Bwaise informal settlement, Kampala, Uganda.

Overall, most participants knew the effects of tobacco use on health. More than three-quarters knew that tobacco causes serious illness, lung cancer and that smokeless tobacco causes serious illness. Less than one-half knew that smoking tobacco causes stroke.

### Factors associated with daily tobacco smoking among the youth in Bwaise informal settlement, Kampala, Uganda.

After controlling for other factors: The prevalence of smoking daily was: 68% higher among males compared to females (adj.PR=1.68 [95%CI=1.27–2.23]), 72% higher among those aged 21–30 years than their younger counterparts (adj.PR=1.72 [95% CI=1.28–2.33]), 36% lower among those whose origin was outside central Uganda (adj.PR=0.64[95%CI=0.46–0.89]), 31% less among those who had secondary education compared to those with primary or no education (adj.PR=0.69 [95% CI=0.56–0.87]), 50% higher among those who didn’t believe that smoking causes serious illness (adj.PR=1.5[95%CI=1.11–2.03]). Participants who didn’t know that smoking causes heart attacks had a 49% higher prevalence of daily smoking compared to their counterparts (adj.PR=1.49 [95% CI=1.11–4.83]), it was similarly 71% higher among participants who didn’t know that smoking causes lung cancer (adj.PR=1.71[CI=1.25–2.35]).

### Factors associated with smokeless tobacco use among the youth in Bwaise informal settlement, Kampala, Uganda.

Overall, the prevalence of smokeless tobacco use was 75% higher among participants aged 21–30 years old compared to their 18–20-year-old counterparts (adj.PR=1.75 [CI=1.04–2.96]). The prevalence of smokeless tobacco use among participants who didn’t believe that smokeless tobacco causes serious illness was 3.02 times that of participants who believed it (adj.PR=3.02 [95%CI=1.87–4.87]).

## Discussion

### Daily Tobacco Smoking

We found that of the 222 current smokers in Bwaise, 72.1% were daily smokers, this accounted for 37.9% of the total sample population. This is consistent with a study among patients living with HIV in which 79.6% were daily smokers (30), and 71% in Kochi, Kerala (31). On the contrary, there were only 10.6% in a STEPS survey in Kenya (32) and 9.2% in another Ugandan study (7). The latter 2 studies were conducted among the general population. Unlike general populations, special populations have a unified attraction towards habits such as smoking (6). In our study, smoking included any type of tobacco product, including shisha, pipes full of tobacco, hand-rolled cigarettes, cigars, and others, not just manufactured cigarettes. Additionally, we had a high number of “*kibanga”* smokers (accounting for 71.3% of daily tobacco smokers). This explains the high prevalence of daily smoking in this study, which implies that future interventions should heavily address the dangers of daily tobacco smoking.

### The commonest type of tobacco product smoked in Bwaise Informal settlement

We found that ‘*kibanga*’ (a mixture of tobacco and marijuana) was the most smoked product daily in our study, unlike conventional literature where manufactured cigarettes are the most smoked tobacco product (7, 30, 33). However, the reasons for using ‘*kibanga’* are scarce. In some studies, it was reported to help with bad moods, coping with stress, relaxation and that it has neutral effects than if each product (tobacco and marijuana) were taken alone (34). In Bwaise,*’kibanga’* is locally hand rolled, hence readily available and affordable by most youth.

### Smokeless Tobacco Use

The prevalence of SLT use was 16.4%, findings on SLT in Uganda are scarce, moreover, the reports available are inconsistent. In a 2013 GATS survey of Uganda, it was 2.4% (35) and 3.6 % in Kenya (32). In a systematic review of 127 countries, it was higher among males (36), in an urban slum in India, it was 27.3% (11). In our study, the high prevalence was predominantly because of the informal nature of the setting, unlike other studies among general populations. Additionally, it was more common among males; anecdotes report that as females grow up; the majority are taken away from such settlements. Moreover, SLT is perceived to be less harmful compared to smoked tobacco (37). The public smoking ban of 2015 in Uganda too could be a contributor to the high SLT use rate in this urban informal setting. SLT forms therefore offer users an opportunity to inhale nicotine without appearing to be smoking in public.

### Factors Associated with daily tobacco smoking

Gender was strongly associated with daily tobacco smoking, this is consistent with other studies in Uganda (7, 35), and in Kenya (32). This has been attributed to the tobacco advertising industry, where men are targeted through constantly depicting tobacco use as a male activity, with benefits such as extraordinary sexual ability and confidence (38–40).

The prevalence of daily tobacco smoking increased with an increase in age. Similar findings were reported in other studies (4, 32, 41). Studies report that most individuals who start smoking rarely stop the habit. The reason for this remains complex, but it has been linked to addiction to nicotine, increasing stress as one grows and peer pressure.

Originating from outside central Uganda was protective against daily smoking. This is contrary to findings from Kabwama et al’s study, (7). The reasons for this are paradoxical given that central Uganda is not predominantly a tobacco-growing region compared to northern or the west Nile regions in Uganda. Self-reports from a previous study revealed that tobacco is a traditional plant in central Uganda, which is used to perform traditional rituals (42). Moreover, the study setting was dominantly occupied by shrines, from which tobacco is directly translated as ‘*taaba*’ meaning to bring together, hence used to reconcile broken relationships.

Attaining higher education was protective against daily tobacco smoking. This is consistent with other findings (7, 32, 41). Education has been labelled as a fundamental of socio-economic circumstances in life (43) so that tobacco use among people with low education levels mirrors a setting honed by stress, strain, economic handicap and poverty, which together amplify tobacco use and dampen cessation attempts (44).

Daily tobacco smoking was higher among individuals who didn’t know that smoking causes lung cancer, heart attack or serious illness. These findings are consistent with those from other studies in Uganda (4) and elsewhere (45–47), which mirrors a function of less perceived threat.

### Factors associated with smokeless tobacco use

SLT increased with age, few studies in Uganda and SSA have assessed the factors associated with SLT. This finding is consistent with that from Kenya (32)The United States and Bangladesh (48, 49). All forms of tobacco contain nicotine, such that once initiated, users get addicted, and as they grow, the habit also grows (3). This implies that tobacco use cessation programs should consider age in their design.

SLT use was 3.02 times among participants who didn’t know that it causes any serious illness compared to those who knew it, consistent with (20) and another study in Bangladesh (50).

SLT is perceived to be less harmful than smoked tobacco (42, 51), however, other studies suggest that both smoked and smokeless tobacco contain nicotine. Based on the health belief model (52), knowledge is linked to perceived threat and cues to action, which mirrors this finding, implying that educational interventions still need to target myths among slum dwellers.

### Study Limitations

The sample size was not big enough to conduct representative subgroup analyses. Tobacco use is self-reported, and therefore subject to social desirability bias.

## Conclusion

i)The majority of youth used smoked tobacco products, the most commonly smoked product was ‘*kibanga*’ while kuber was the most common smokeless tobacco product used. Daily tobacco smokers were 37.9% of the total sample population while 16.4% used smokeless tobacco. ii) The majority of participants knew the effects of tobacco use on health. iii) Daily tobacco smoking is associated with gender, age, education level, region of origin and knowledge that tobacco smoking causes serious illness, lung cancer or heart attacks in this setting. iv) Smokeless tobacco use is associated with age and knowledge of whether smokeless tobacco causes serious illness and heart attacks or not.

### Policy implications

To support tobacco control among the youth, current and future policy interventions should mostly target males aged 21 years and above, of education level below secondary, and those who don’t believe that daily tobacco smoking and smokeless tobacco use can lead to heart attacks, lung cancer and serious illnesses. Health education policies and packages in informal communities should address the myths around the use *of ‘kibanga’* while discouraging the use of other common forms of tobacco. The Ministry of Health and other partners should devise means that employ a mix of communication channels when distributing anti-tobacco messages, this will cater for the majority with primary or no education at all.

### Recommendations

1. Other studies in a similar setting can be conducted using larger sample sizes to enable stronger subgroup analyses.
2. Reasons for the popular use of ‘*kibanga*’ in this setting need to be further explored.

## Figures and Tables

**Figure 1:**
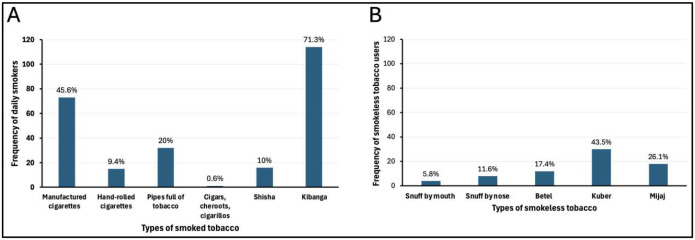
Types of tobacco products used by the youth in Bwaise: smoked tobacco products (A) and smokeless tobacco products (B)

**Table 1:** Characteristics of the study participants

Characteristic	Number of respondents (n)	Proportion (%)
**Gender**		
Male	248	58.8
Female	174	41.2
**Age, Mean (SD)**	**23(±3.64)**	
18–20years	123	29.2
21–30years	299	70.8
**Marital status**		
Not married	314	74.4
Married	108	25.6
**Region of origin**		
Central	305	72.3
Eastern	41	9.7
Western	58	13.7
Others	18	4.3
**Education level**		
None/primary	137	32.5
Secondary/University	285	67.5
**Religion**		
Catholic	109	25.8
Protestant	61	14.5
Pentecostal	43	10.2
Adventist	13	3.0
Moslem	192	45.5
Other	4	1.0
**Employment status**		
Employed	349	82.7
Unemployed	73	17.3
**Monthly income**		
less than 250,000/=	188	44.6
250,000–500,000/=	106	25.1
More than 500,000/=	77	18.3
Don’t know	51	12.1

**Table 2:** Tobacco use patterns of the study participants by gender

	Gender	
Tobacco use pattern	Male n(%)	Females n(%)
Ever smoker		
Yes	208 (68.7)	94 (31.13)
No	40 (33.3)	80 (66.7)
Current smoker		
Yes	158 (71.2)	68 (28.8)
No	90 (45.0)	110 (55)
***Current daily smoker (n=222)***		
***Yes***	***118 (73.8)***	***42 (26.2)***
***No***	***40 (64.5)***	***22 (35.5)***
Made previous quit attempts (n=222)		
Yes	91 (71.1)	37 (28.9)
No	67 (71.3)	27 (28.7)
Received quit advice previously (n=222)		
Yes	23 (63.9)	13 (36.1)
No	44 (66.7)	22 (33.3)
No visit during the past months	91 (75.8)	29 (24.2)
Past smoker		
Yes	50 (62.5)	30 (37.5)
No	40 (33.3)	80 (66.7)
***Current smokeless tobacco user***		
***Yes***	***48 (69.6)***	***21(30.4)***
***No***	***200 (56.7)***	***153 (43.3)***
Current daily smokeless tobacco user		
Yes	30 (68.2)	14 (31.8)
No	18 (72.0)	7 (28.0)
Ever smokeless tobacco user		
Yes	95 (75.4)	31 (24.6)
No	153 (51.7)	143 (48.3)
Ever daily smokeless tobacco user		
Yes	44 (83.0)	9 (17.0)
No	51 (70.0)	22 (30.0)
Smoking in the home (past 30 days)		
Yes	120 (61.9)	74 (38.1)
No	128 (56.1)	100 (43.9)
Smoking in the workplace (past 30 days)		
Yes	196 (60.9)	126 (39.1)
No	38 (61.3)	24 (38.7)
Don’t work in a closed area	14 (36.8)	24 (63.2)

In bold and italicized are the main patterns of focus in this study. Daily tobacco smoking = 160/222, which accounts for 160/422(37.9%) of the entire sample population. Smokeless tobacco use=69/422(16.4%).

**Table 3:** Knowledge of the effects of tobacco use on health among the study participants.

Knowledge item	Number of respondents (n)	Proportion (%)
**Knows that smoking causes:**		
**Serious illness**		
Yes	369	87.4
No	30	7.1
Don’t know	23	5.5
**Stroke**		
Yes	189	44.8
No	85	20.1
Don’t know	148	35.1
**Heart attack**		
Yes	278	65.9
No	64	15.1
Don’t know	80	19.0
**Lung cancer**		
Yes	382	90.5
No	14	3.3
Don’t know	26	6.2
**Knows that smokeless tobacco causes:**		
**Serious illness**		
Yes	305	72.3
No	41	9.7
Don’t know	76	18.0

*To assess knowledge; frequencies and percentages were computed for each knowledge item.

**Table 4:** Multivariate analysis of factors associated with daily tobacco smoking.

Variable	Yes (N=160)	No (N=262)	Unadjusted estimates	Adjusted estimates
	n (%)	n (%)	PR (95% CI)	PR (95% CI)
**Gender**				
Female	118(47.6)	130(52.4)	1	1
Male	42(24.1)	132(75.9)	**1.97(1.47–2.65)** ***	**1.68(1.27–2.23)** ***
**Age**				
18–20years	31(25.2)	92(74.8)	1	1
21–30years	129 (43.1)	170(56.9)	**1.71(1.23–2.38)** **	**1.72(1.28–2.33)** ***
**Marital status**				
Not married	118(37.7)	195(62.3)	1	----
Married	42(38.5)	67(61.5)	1.02(0.77–1.35)	
**Region of origin**				
Central	132(43.8)	173(56.7)	1	1
Outside Central	89(76.1)	28(23.9)	**0.55(0.39–0.78)** **	**0.64(0.46–0.89)** *
**Education level**				
None/primary	67(48.9)	70(51.1)	1	1
Secondary/University	93(32.7)	191(67.3)	**0.67(0.53–0.85)** **	**0.69(0.56–0.87)** **
**Religion**				
Christian	83(36.1)	147(63.9)	1	----
Non-Christian	77(40.1)	115(59.9)	1.11(0.87–1.42)	
**Employment status**				
Employed	145(41.5)	204(58.5)	1	----
Unemployed	15(20.5)	58(79.5)	0.49(0.31–0.79) **	----
**Monthly income**				
Above 500,000/=	21(27.3))	56(72.7)	1	----
less than 250,000/=	81(43.1)	107(56.9)	1.56(0.69–1.23) *	----
250,000–500,000/=	42(39.6)	64(60.4)	1.5(0.29–1.09)	----
Don’t know	16(31.4)	35(68.6)	1.2(0.47–1.13)	----
**Knows that smoking causes:**				
**Serious illness**				
Yes	123(33.3)	246(66.7)	1	1
No	24(80.0)	6(20.0)	**2.4(1.91–3.02)** ***	**1.5(1.11–2.03)** **
Don’t know	13(56.5)	10(43.5)	1.69(1.15–2.50) *	1.18(0.78–1.77)
**Stroke**				
Yes	65(34.4)	124(65.6)	1	----
No	46(54.1)	39(45.9)	1.57(1.56–2.62) **	----
Don’t know	49(33.1)	99(66.9)	1.37(1.01–1.87)	----
**Heart attack**				
Yes	86(30.95)	192(69.1)	1	1
No	40(62.5)	24(37.5)	**2.02(1.56–2.62)** ***	**1.49(1.11–4.83)** *
Don’t know	34(42.5)	46(57.5)	**1.37(1.01–1.87)** *	1.09(0.81–1.47)
**Lung cancer**				
Yes	129(33.8)	253(66.2)	1	1
No	12(85.7)	2(14.3)	**2.54(1.96–3.27)** ***	1.23(0.88–1.73)
Don’t know	19(73.1)	7(26.9)	**2.16(1.65–2.84)** ***	**1.71(1.25–2.35)** **
**Knows smokeless tobacco causes serious illness**				
Yes	105(34.4)	200(65.6)	1	----
No	20(48.8)	21(51.2)	1.42(0.99–2.01) *	----
Don’t know	35(46.1)	41(53.9)	1.34(1.00 –1.78) *	----

* Statistically significant at 5% level of significance, *p<0.05,

** p<0.01,

*** p<0.001.

PR = Prevalence Ratio, CI = Confidence Interval. Stroke was not significant in the multivariate model so not included, the last knowledge item was not included in the final model because of its questioned plausibility to tobacco smoking.

**Table 5:** Multivariate analysis of factors associated with Smokeless Tobacco use.

Variable	Yes (N=69)	No (N=353)	Unadjusted estimates	Adjusted estimates
	n(%)	n(%)	PR (95% CI)	PR (95% CI)
**Gender**				
Female	48(19.4)	200(80.6)	**1**	1
Male	21(12.1)	153(87.9)	**1.60(0.99–2.58)** *	1.45(0.91–2.31)
**Age**				
18–20years	14(19.4)	109(88.6)	1	**1**
21–30years	55(18.4)	244(81.6)	1.62(0.93–2.79)	**1.75(1.04–2.96)** *
**Marital status**				
Not married	54(17.3)	259(82.7)	1	----
Married	15(13.8)	94(86.2)	0.79(0.47–1.35)	
**Region of origin**				
Central	53(17.4)	252(82.6)	1	----
Outside Central Uganda	16(13.7)	101(86.3)	0.79(0.47–1.32)	
**Education level**				
None/primary	25(18.3)	112(81.7)	1	1
Secondary/University	44(15.5)	240(84.5)	0.85(0.54–1.33)	0.89(0.57–1.40)
**Religion**				
Christian	38(16.5)	192(83.5)	1	----
Non-Christian	31(16.2)	161(83.8)	0.98(0.63–1.51)	
**Employment status**				
Employed	62(17.8)	287(82.2)	1	----
Unemployed	7(9.6)	66(90.4)	0.54(0.26–1.13)	
**Monthly income**				
Above 500,000/=	10(13.0)	67(87.0)		
Less than 250,000/=	31(16.5)	157(83.5)	1.3(0.65–2.46)	
250,000–500,000/=	21(19.8)	85(80.2)	1.5(0.76–1.92)	
Don’t Know	7(13.7)	44(86.3)	1.1(0.43–2.59)	
**Knows that smoking causes:**				
**Serious illness**				
Yes	59(16.0)	310(84.0)	1	----
No	6(20.0)	24(80.0)	1.25(0.59–2.66)	
Don’t know	4(82.6)	19(17.4)	1.09(0.43 –2.73)	
**Stroke**				
Yes	33(17.5)	156(82.5)	1	----
No	16(18.8)	69(81.2)	1.08(0.63–1.85)	
Don’t know	20(13.5)	128(86.5)	0.77(0.46–1.29)	
**Heart attack**				
Yes	45(16.2)	233(83,8)	1	1
No	8(12.5)	56(87.5)	0.77 (0.38–1.56)	0.56(0.28–1.1) *
Don’t know	16(20.0)	64(80.0)	1.23(0.74–2.07)	0.74(0.77–2.1)
**Lung cancer**				
Yes	60(15.7)	322(84.3)	1	----
No	4(28.6)	10(71.4)	1.82(0.77–4.30)	
Don’t know	5(19.2)	21(80.8)	1.22(0.53–2.79)	
**Knows smokeless tobacco causes serious illness**				
Yes	44(14.4)	261(85.6)	**1**	**1**
No	17(41.5)	24(58.5)	**2.87(1.82–4.53)** ***	**3.02(1.87–4.87)** ***
Don’t know	8(10.5)	68(89.5)	0.73(0.36 –1.49)	----

* Statistically significant at 5% level of significance, *p<0.1,

** p<0.05,

*** p<0.001.

PR = Prevalence Ratio, CI = Confidence Interval. Age, gender, and education level were left in the model because of their plausibility with tobacco use. When re-introduced, heart attack became significant

## Data Availability

The dataset used and/or analysed during the current study are available from the corresponding author on request

## List of abbreviations

AIC: Akaike Information Criteria
APR: Adjusted Prevalence Ratio
BIC: Bayesian Information Criteria
CADCA: Community Anti-Drug Coalitions of America
CDC: Centers for Disease Control and Prevention
CPR: Crude Prevalence Ratio
CTCA: Centre for Tobacco Control in Africa
GATS: Global Adult Tobacco Survey
GYTS: Global Youth Tobacco Survey
MOH: Ministry of Health
NCD: Non-Communicable Disease
PR: Prevalence Ratio
SLT: Smokeless Tobacco
SSA: Sub-Saharan Africa
UDHS: Uganda Demographic Health Survey
UHHS: Uganda Household Survey
VIF: Variance Inflation Factor
WHO: World Health Organization
